# Dolutegravir-Associated Dyslipidemia and Atherogenic Lipid Changes Among Treatment-Naïve People Living With HIV: A 12-Month Prospective Cohort Study From Southern India

**DOI:** 10.7759/cureus.111987

**Published:** 2026-07-03

**Authors:** Savitha A Sebastian, Cerin Devasia, Sumithra Selvam, Jyothi Idiculla

**Affiliations:** 1 Internal Medicine, St. John’s Medical College Hospital, Bengaluru, IND; 2 Internal Medicine, St. Thomas Hospital, Changanassery, IND; 3 Biostatistics, St. John’s Research Institute, Bengaluru, IND

**Keywords:** antiretroviral agents, antiretroviral therapy, cholesterol, dolutegravir, dyslipidemias, hiv integrase inhibitors, india

## Abstract

Background

Dolutegravir (DTG)-based antiretroviral therapy (ART) is widely used for the treatment of people living with HIV (PLHIV). As cardiovascular disease risk becomes increasingly important in the long-term management of PLHIV, understanding changes in lipid parameters following initiation of DTG-based therapy is clinically relevant. Prospective data on dyslipidemia and atherogenic lipid indices among Indian PLHIV initiating tenofovir disoproxil fumarate-lamivudine-dolutegravir (TLD) remain limited. This study aimed to evaluate changes in lipid profiles and atherogenic indices over 12 months among ART-naïve PLHIV initiating TLD.

Methodology

We conducted a 12-month prospective cohort study at the HIV Clinic of a tertiary teaching hospital in Bengaluru, India. A total of 70 adult ART-naïve PLHIV initiating TLD were enrolled consecutively. Participants with baseline dyslipidemia, lipid-lowering therapy, pregnancy, or lactation were excluded. Fasting lipid profile, including serum total cholesterol (TC), low-density lipoprotein cholesterol (LDL-c), high-density lipoprotein cholesterol (HDL-c), triglycerides (TG), and atherogenic indices (TC/HDL-c ratio, TG/HDL-c ratio, non-HDL cholesterol) were measured at baseline and 12 months. Body mass index (BMI), fasting blood sugar (FBS), serum alanine aminotransferase (ALT), and serum creatinine were also recorded at these time points. Enrolled participants were followed up for 12 months to determine the one-year incidence of dyslipidemia and changes in lipid parameters and atherogenic indices.

Results

All 70 participants completed follow-up (100% retention; median age = 33 years (interquartile range = 27-40); 74.3% male). The main finding was that 24/70 (34.29%, 95% confidence interval = 23.5-46.7) developed incident dyslipidemia at 12 months, with 17/24 (70.8%) demonstrating ≥2 concurrent lipid abnormalities. Low HDL-c (24.29%) and hypertriglyceridemia (22.86%) were most frequent. Statistically significant adverse mean changes occurred in TC (157.1 → 164.6 mg/dL; t(69) = 2.23, p = 0.029), LDL-c (95.8 → 111.1 mg/dL; t(69) = 5.15, p < 0.001), TG (131.7 → 154.7 mg/dL; Z = 3.60, p < 0.001), non-HDL-c (108.9 → 119.8 mg/dL; Z = 2.45, p = 0.014), TC/HDL-c ratio (3.37 → 4.03; Z = 2.18, p = 0.029), and TG/HDL-c ratio (2.85 → 4.04; Z = 3.05, p = 0.002). HDL-c declined non-significantly (48.2 → 44.8 mg/dL; t(69) = -1.83, p = 0.071). BMI rose +0.67 kg/m² (t(69) = 3.43, p = 0.001), serum creatinine +0.11 mg/dL (t(69) = 4.86, p < 0.001), ALT increased by 12.6 IU/L (Z = -5.97, p < 0.001), and FBS +7.6 mg/dL (t(69) = 2.00, p = 0.049), without new diabetes.

Conclusions

DTG-based antiretroviral therapy (TLD) was associated with a 34.29% one-year incidence of dyslipidemia in ART-naïve Indian PLHIV, with significant adverse shifts in atherogenic indices. These findings support routine lipid and metabolic surveillance and consideration of statin therapy in eligible patients as essential components of monitoring care for PLHIV on DTG-based ART.

## Introduction

Combination antiretroviral therapy (cART) has transformed HIV infection from a fatal disease into a manageable chronic condition, with consequent normalization of life expectancy in people living with HIV (PLHIV). This shift has translated into a corresponding rise in age-associated non-communicable comorbidities, most notably cardiovascular disease (CVD). CVD is now one of the leading non-AIDS causes of mortality in PLHIV, and dyslipidemia, driven by viral inflammation and antiretroviral toxicity, is its dominant modifiable risk factor [1].

Against this backdrop, dolutegravir (DTG), a second-generation integrase strand transfer inhibitor (INSTI), has become the cornerstone of first-line cART globally following the 2018 World Health Organization (WHO) recommendation that all PLHIV initiate DTG-based regimens [2]. The Indian National AIDS Control Organisation (NACO) adopted tenofovir disoproxil fumarate-lamivudine-dolutegravir (TLD) as the preferred first-line antiretroviral therapy (ART) in 2021 [3].

Compared with efavirenz, DTG demonstrates superior virological efficacy, a higher genetic barrier to resistance, fewer neuropsychiatric adverse effects, and improved tolerability, which have driven its rapid global uptake [4,5]. The metabolic safety of DTG, however, has been under increasing scrutiny. Pooled analyses of randomized controlled trials have shown that INSTI-based regimens, particularly DTG and bictegravir, are associated with greater weight gain than non-nucleoside reverse transcriptase inhibitor (NNRTI) or protease inhibitor (PI)-based regimens, with mean differences of approximately 2.0 to 6.0 kg over 48-96 weeks [6]. In the ADVANCE trial from South Africa, mean weight gain at 96 weeks reached 7.1 kg in the DTG plus tenofovir alafenamide (TAF)/emtricitabine arm and 4.3 kg in the DTG plus tenofovir disoproxil fumarate (TDF)/emtricitabine arm, compared with 2.3 kg with the efavirenz arm. Female sex, Black ancestry, lower baseline CD4 count, and higher baseline viral load have been consistently identified as predictors of excess weight gain [7]. A recent Ghanaian prospective cohort reported a metabolic syndrome incidence of 384.2 per 1,000 person-years following DTG initiation [8]. The mechanism of these metabolic effects appears to be multifactorial: in vitro evidence indicates that DTG induces adipose tissue fibrosis and insulin resistance and suppresses adiponectin secretion [9].

The lipid effects of DTG are more nuanced. In direct comparisons with boosted PIs and efavirenz, DTG generally produces smaller increases in total cholesterol (TC), low-density lipoprotein cholesterol (LDL-c), and triglycerides (TG) [10]. Nevertheless, the RESPOND consortium, a large prospective European cohort of 4,577 PLHIV, reported an overall incidence of dyslipidemia of 191.6 per 1,000 person-years. Compared with boosted PIs, dyslipidemia associated with DTG was favorable (adjusted incidence rate ratio (aIRR) = 0.71), but it was higher than that associated with NNRTIs (aIRR = 1.35) [11]. Real-world observational data from sub-Saharan Africa have documented a striking pattern of low high-density lipoprotein cholesterol (HDL-c) predominance; in a Zambian cohort, 75% of young adults on DTG-based regimens had low HDL-c [12].

The REPRIEVE trial has heightened the clinical urgency of characterizing the lipid effects associated with DTG. It was a randomized, double-blind, placebo-controlled study of 7,769 PLHIV at low-to-moderate cardiovascular risk that was stopped early in 2023 after demonstrating a 35% reduction in major adverse cardiovascular events with pitavastatin 4 mg daily (hazard ratio = 0.65, 95% confidence interval (CI) = 0.48-0.90) [13]. REPRIEVE has since informed the 2026 American College of Cardiology (ACC)/American Heart Association (AHA) Joint Committee guideline on the management of dyslipidemia. Updated guidelines recommend moderate-intensity statin therapy for all PLHIV aged 40-75 years, irrespective of baseline LDL-c [14]. However, the trial was conducted primarily before the global rollout of DTG-based first-line ART in low- and middle-income countries (LMICs), leaving open the question of how an INSTI-based regimen affects lipid profiles in this population.

Despite the rapid scale-up of DTG-based ART in India under the NACO program since 2020, prospective data on dyslipidemia among PLHIV initiating DTG in India remain sparse. Dravid et al. conducted a multi-center Phase IV trial of TLD in 250 treatment-naïve Indian patients, reporting lipid changes during routine 24-week safety monitoring, without specific quantification of the incidence of dyslipidemia or stratification by lipid fraction [15]. Joshi et al., in a six-month prospective observational study of 319 PLHIV in Mumbai, documented statistically significant increases in TC and TG following DTG initiation, with a 14.4% prevalence of hyperlipidemia; however, 92.5% of their cohort were ART-experienced switchers from prior NNRTI- or PI-based regimens [16]. Sekar et al. reported dyslipidemia as the second most common adverse drug reaction in a six-month observational study of 97 PLHIV, with DTG identified as the most frequently implicated agent, but again in a predominantly switch population [17]. To our knowledge, few Indian studies have prospectively quantified the one-year incidence of dyslipidemia in ART-naïve PLHIV initiating DTG-based therapy, characterized lipid fractions, or reported atherogenic indices such as TC/HDL-c, TG/HDL-c, and non-HDL-c. This leaves an important gap in 12-month prospective data from ART-naïve Indian PLHIV initiating DTG-based therapy.

Therefore, to build on available data from India, we conducted a 12-month prospective cohort study to determine incident dyslipidemia, the magnitude of change in individual lipid parameters and atherogenic indices (TC/HDL-c ratio, TG/HDL-c ratio, non-HDL-c), and the broader metabolic safety profile (weight, body mass index (BMI), renal and hepatic indices) in adult PLHIV initiated on DTG-based cART at a tertiary referral center in southern India.

## Materials and methods

Study design and setting

This prospective cohort study was conducted at the HIV Clinic of a tertiary teaching institution in Bengaluru, India. The clinic caters predominantly to a socially and economically disadvantaged population of PLHIV and provides comprehensive HIV services, including free ART through NACO. Participants were enrolled and followed between April 2023 and October 2024. The protocol was approved by the Institutional Ethics Committee of St. John’s Medical College (reference number: TH-112/2023; dated April 4, 2023) and conducted in accordance with the Declaration of Helsinki and the Indian Council of Medical Research (ICMR) National Ethical Guidelines for Biomedical Research on Human Participants. All participants provided written informed consent before enrolment. Participants were followed prospectively for 12 months, with data collected at baseline and at the 12-month follow-up visit.

Participants

Adults (≥18 years) with confirmed HIV infection who were newly initiated on a DTG-based cART regimen at the clinic were enrolled consecutively. The standard regimen used at the center, in alignment with NACO guidelines, was TDF 300 mg/lamivudine (3TC) 300 mg/dolutegravir (DTG) 50 mg (TLD) as a once-daily fixed-dose combination. Participants were excluded if they were currently receiving lipid-lowering therapy, had baseline dyslipidemia per the National Cholesterol Education Program Adult Treatment Panel III (ATP III) criteria [18], were pregnant or lactating, or were unable to provide informed consent.

Procedures and laboratory assessment

All participants underwent a structured baseline evaluation at enrolment, including demographic and clinical history, comorbidity assessment, anthropometry (height, weight, calculated BMI), and laboratory investigations (12-hour fasting lipid profile: TC, TG, HDL-c, LDL-c; fasting blood sugar, serum alanine aminotransferase (ALT), and serum creatinine). Anthropometric measurements were obtained with participants barefoot and wearing light clothing. Standing height was measured using a stadiometer (Seca, Germany) to the nearest 0.5 cm, while body weight was measured using a calibrated digital weighing scale (Omron, India) to the nearest 0.1 kg. Participants were reassessed at 12 months using identical clinical and laboratory procedures. Data were prospectively captured using a structured proforma and entered electronically into EpiCollect5.

All laboratory investigations were performed at the NABL-accredited laboratory of St. John’s Medical College Hospital, Bengaluru. Serum TC was measured using an enzymatic cholesterol oxidase-peroxidase method. HDL-c was measured using an accelerator selective detergent method with cholesterol oxidase/esterase. LDL-c was measured directly using a liquid selective detergent method with cholesterol oxidase/esterase. Serum TG was measured using the glycerol phosphate oxidase enzymatic method. All lipid parameters were analyzed on the Abbott Architect platform (Abbott Laboratories, Abbott Park, IL, USA).

Definitions, outcomes, and variables

The primary outcome was incident dyslipidemia at 12 months. Incident dyslipidemia was diagnosed in any participant who, at the 12-month visit, fulfilled at least one of the following criteria adapted from the ATP III guidelines: TC ≥240 mg/dL; HDL-c <40 mg/dL; LDL-c ≥160 mg/dL, and TG ≥200 mg/dL [18].

Secondary outcomes included the magnitude of change in each lipid fraction; in derived atherogenic indices (TC/HDL-c ratio, TG/HDL-c ratio, non-HDL-c (calculated as TC - HDL-c)); and in metabolic safety parameters (weight, BMI, FBS, ALT, serum creatinine).

The independent variables were age, sex, smoking, alcohol use, and change in BMI. BMI was categorized as underweight (<18.5 kg/m²), normal (18.5-22.9 kg/m²), overweight-at-risk (23.0-24.9 kg/m²), obese class I (25.0-29.9 kg/m²), and obese class II (≥30.0 kg/m²) per WHO Asia-Pacific criteria [19].

Sample size estimation

Sample size was calculated a priori for the primary outcome, i.e., the one-year incidence of dyslipidemia in ART-naïve PLHIV initiated on DTG-based cART. The expected proportion from a comparable LMIC population: Fiseha et al. reported a dyslipidemia prevalence of 59.9% (95% CI = 55.0-64.7%) among 392 Ethiopian PLHIV on dolutegravir-based cART [20]. Given the absence of prospective Indian incidence data for DTG-based ART at the time of study design, we adopted a conservative expected proportion of 50% and targeted an absolute precision of ±12% at the 95% confidence level. Using the single-proportion formula, the minimum required sample size was 67 participants, inflated to 70 to allow for a 5% margin for loss to follow-up.

Statistical analysis

Statistical analyses were performed using SPSS Statistics version 29.0 (IBM Corp., Armonk, NY, USA). Continuous variables are presented as mean ± standard deviation (SD) or median (interquartile range (IQR)), as appropriate. Categorical variables are presented as frequencies and percentages. Changes in continuous lipid and metabolic parameters between baseline and 12 months were assessed using paired t-tests or the Wilcoxon signed-rank test, as appropriate. Associations between changes in weight and BMI and changes in lipid profile parameters were evaluated using Spearman’s rank correlation coefficient. The associations between age and sex and dyslipidemia were assessed using the chi-square test and Fisher’s exact test, respectively. All statistical tests were two-sided, and p-values <0.05 were considered statistically significant.

## Results

Baseline participant characteristics

In total, 70 adult PLHIV were consecutively enrolled, and all completed the 12-month follow-up (100% retention). The median age was 33 years (IQR = 27-40; range = 18-61), and the majority (n = 52; 74.3%) were male. Hypertension was present in five (7.1%) participants, diabetes mellitus in one (1.4%), and hepatitis B co-infection in two (2.8%); 12 (17.1%) participants reported alcohol use, and six (8.6%) were active smokers. The mean baseline weight was 63.8 ± 12.5 kg, and the mean BMI was 23.09 ± 3.84 kg/m². By WHO Asia-Pacific criteria, 10 (14.3%) participants were underweight, 20 (28.6%) had normal BMI, 14 (20.0%) were overweight-at-risk, 23 (32.9%) were obese class I, and three (4.3%) were obese class II. The mean baseline FBS was 95 ± 14 mg/dL, and the mean baseline ALT was 30.3 ± 17.4 IU/L. No participant met any ATP III criterion for dyslipidemia at baseline.

Incidence and pattern of dyslipidemia at one year

**Figure 1:**
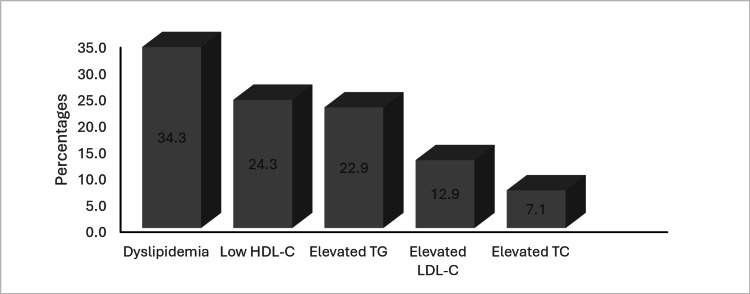
Pattern of incident dyslipidemia at 12 months in PLHIV on DTG-based cART. Pattern of incident dyslipidemia at 12 months in ART-naïve PLHIV on dolutegravir-based cART (n = 70). Bars show the proportion meeting each ATP III diagnostic threshold. Categories are non-mutually exclusive; 47 lipid abnormalities occurred across 24 affected participants. HDL-C = high-density lipoprotein cholesterol; LDL-C = low-density lipoprotein cholesterol; TC = total cholesterol; TG = triglycerides; PLHIV = people living with HIV; DTG = dolutegravir; cART = combination antiretroviral therapy; ART = antiretroviral therapy; ATP III = Adult Treatment Panel III

At the 12-month follow-up visit, 24 of 70 participants (34.29%, 95% CI = 23.5-46.7%) had developed incident dyslipidemia. The most frequent component was low HDL-c (<40 mg/dL), present in 17 (24.29%) participants, followed by hypertriglyceridemia (≥200 mg/dL) in 16 (22.86%) participants, elevated LDL-c (≥160 mg/dL) in nine (12.86%) participants, and elevated TC (≥240 mg/dL) in five (7.14%) participants. Multiple lipid abnormalities frequently co-occurred: of the 24 participants with incident dyslipidemia, seven (29.2%) had a single abnormal lipid parameter, 12 (50.0%) had two concurrent abnormalities, four (16.7%) had three, and one (4.2%) met all four diagnostic criteria simultaneously. Thus, 17 of 24 affected participants (70.8%) demonstrated at least two concurrent lipid abnormalities, indicating a mixed dyslipidemic phenotype rather than isolated single-parameter perturbation. The pattern of incident dyslipidemia is depicted in Figure 1.

Changes in lipid parameters and atherogenic indices

**Figure 2:**
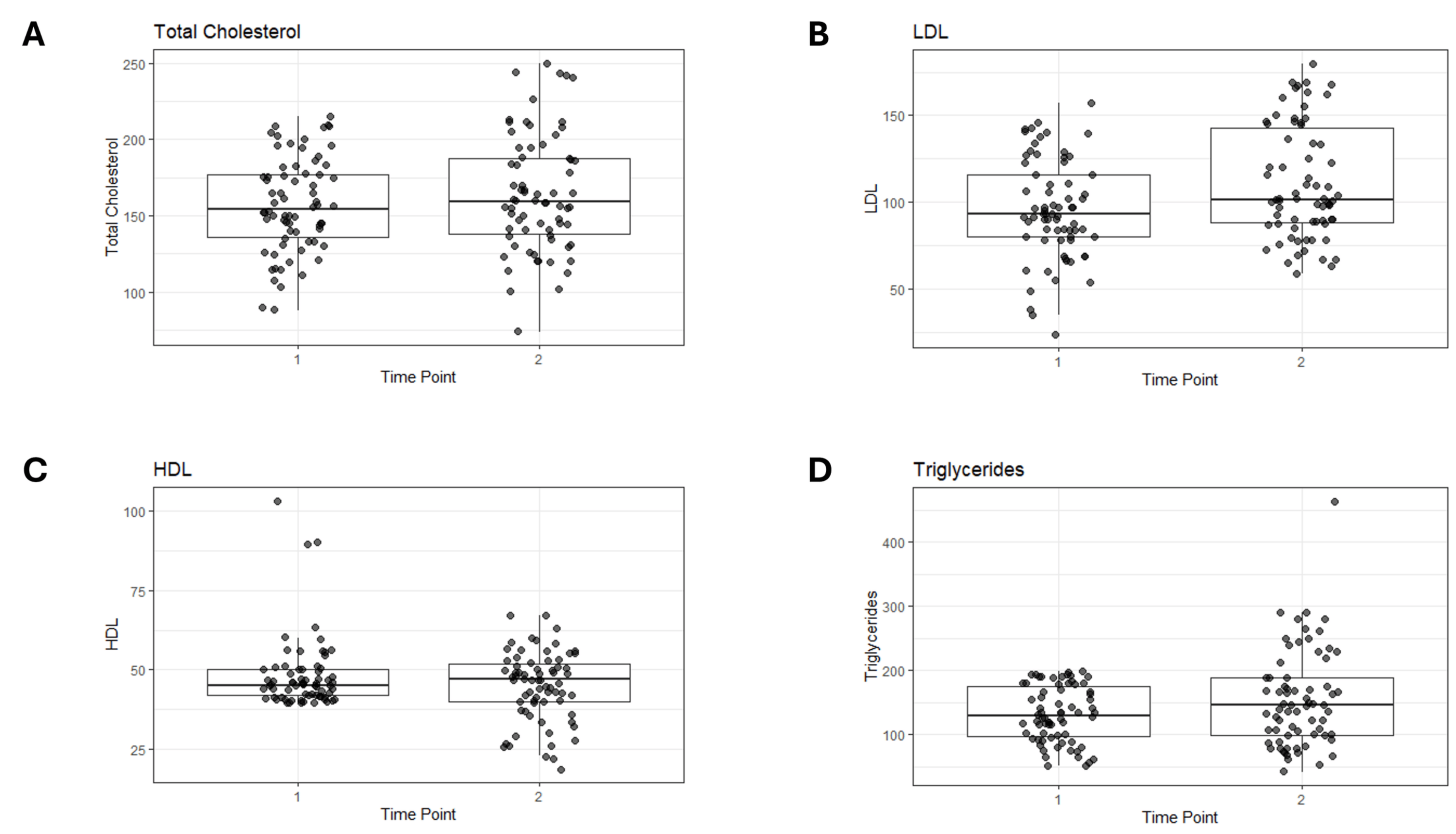
Distribution of fasting lipid parameters at baseline and at 12 months in PLHIV initiated on TLD (n = 70). Panels show distributions for (A) total cholesterol, (B) LDL-cholesterol, (C) HDL-cholesterol, and (D) triglycerides, expressed in mg/dL. Box plots display the mean (horizontal line within the box), interquartile range (box); overlaid jittered points represent individual participant values. All measurements were obtained from 12-hour fasting samples. Significant increases were observed in mean total cholesterol (157.1 → 164.6 mg/dL; t(69) = 2.23, p = 0.029), mean LDL-cholesterol (95.8 → 111.1 mg/dL; t(69) = 5.15, p < 0.001), and mean triglycerides (131.7→154.7 mg/dL; Z = 3.60, p < 0.001). Mean HDL-cholesterol declined from 48.2 to 44.8 mg/dL but did not reach statistical significance (t(69) = -1.83, p = 0.071). PLHIV = people living with HIV; TLD = tenofovir disoproxil fumarate - lamivudine - dolutegravir; LDL = low-density lipoprotein; HDL = high-density lipoprotein

We observed statistically significant adverse changes in all major lipid parameters, except HDL-c, which narrowly missed statistical significance. Mean TC increased from 157.1 ± 30.8 mg/dL to 164.6 ± 38.4 mg/dL (mean increase = +7.5 mg/dL; t(69) = 2.23, p = 0.029). Mean LDL-c rose from 95.8 ± 28.7 mg/dL to 111.1 ± 32.4 mg/dL (mean increase = +15.3 mg/dL; t(69) = 5.15, p < 0.001). Mean TG increased from 131.7 ± 43.3 mg/dL to 154.7 ± 75.1 mg/dL (mean increase = +23.0 mg/dL; Z = 3.60, p < 0.001). Mean HDL-c declined from 48.2 ± 11.3 mg/dL to 44.8 ± 10.9 mg/dL (mean decrease = −3.4 mg/dL; t(69) = -1.83, p = 0.071). Figure 2 shows the distribution of individual lipid parameters.

Atherogenic indices showed statistically significant adverse shifts. Mean non-HDL-c rose from 108.9 ± 32.3 mg/dL to 119.8 ± 43.5 mg/dL (Z = 2.45, p = 0.014). Mean TC/HDL-c ratio increased from 3.37 ± 0.82 to 4.03 ± 1.77 (Z = 2.18, p = 0.029), and mean TG/HDL-c ratio rose from 2.85 ± 1.06 to 4.04 ± 3.02 (Z = 3.05, p = 0.002). Lipid changes are summarized in Table 1.

**Table 1:** Changes in lipid parameters and atherogenic indices in PLHIV initiated on TLD at 12 months (n = 70). Variables are presented as mean ± SD. Comparisons were performed using paired t-tests or Wilcoxon signed-rank tests as appropriate. #: Statistically significant at p < 0.05; *: Wilcoxon signed-rank test. PLHIV = people living with HIV; TLD = tenofovir disoproxil fumarate - lamivudine - dolutegravir; LDL-c = low-density lipoprotein cholesterol; HDL-c = high-density lipoprotein cholesterol; TC = total cholesterol; TG = triglycerides; CI = confidence interval

Parameter	Baseline mean (SD)	12-month mean (SD)	Change from baseline (95% CI)	Test statistic	P-value
Total cholesterol (mg/dL)	157.1 (30.8)	164.6 (38.4)	+7.5 (0.8–14.2)	t = 2.23	0.029^#^
LDL-c (mg/dL)	95.8 (28.7)	111.1 (32.4)	+15.3 (9.4–21.2)	t = 5.15	<0.001^#^
HDL-c (mg/dL)	48.2 (11.3)	44.8 (10.9)	−3.4 (−7.0–0.3)	t = -1.83	0.071
Triglycerides (mg/dL)*	131.7 (43.3)	154.7 (75.1)	+23.0 (8.5–37.5)	Z = 3.60	<0.001^#^
Non-HDL cholesterol (mg/dL)*	108.9 (32.3)	119.8 (43.5)	+10.8 (2.1–19.6)	Z = 2.45	0.014^#^
TC/HDL-c ratio*	3.37 (0.82)	4.03 (1.77)	+0.66 (0.2–1.1)	Z = 2.18	0.029^#^
TG/HDL-c ratio*	2.85 (1.06)	4.04 (3.02)	+1.19 (0.5–1.8)	Z = 3.05	0.002^#^

Baseline-to-follow-up category shifts

Category-shift analysis from baseline to 12 months supported the directional findings of the continuous measures. For TC, four of the eight participants (50%) with baseline borderline-high TC (200-239 mg/dL, below the ATP III diagnostic threshold of ≥240 mg/dL) progressed to elevated TC (≥240 mg/dL) at 12 months. Among the 44 participants with optimal baseline LDL-c (<100 mg/dL), 16 (36.4%) shifted to a worse category, including one (2.3%) who progressed directly to high LDL-c (≥160 mg/dL). For HDL-c, of the six participants with baseline cardioprotective HDL-c (≥60 mg/dL), four (66.7%) regressed to the normal range (40-59 mg/dL), and one (16.7%) further declined to low HDL-c (<40 mg/dL). For triglycerides, 12 of the 45 participants (26.7%) with baseline normal TG (<150 mg/dL) progressed to the borderline-high (150 - 199 mg/dL) or high TG (200 - 499 mg/dL) categories. Bidirectional movement was observed, with a smaller proportion of participants showing improvement in TC (n = 4), LDL-c (n = 4), HDL-c (n = 3), and TG (n = 7) over the 12 months; however, the net direction of movement was unfavorable across all four parameters. These category transitions confirm that the observed mean changes represent true clinical progression rather than statistical drift around baseline values.

Metabolic safety: anthropometric, renal, hepatic, and glycemic parameters

Over 12 months, mean body weight increased from 63.8 ± 12.5 kg to 65.7 ± 13.1 kg (mean increase = 1.89 kg, 95% CI = 0.79-2.98; t(69) = 3.44, p = 0.001), and 30% of participants experienced a weight increase of ≥5% from baseline. Mean BMI rose significantly from 23.09 ± 3.84 to 23.76 ± 3.94 kg/m² (mean increase = +0.67 kg/m²; t(69) = 3.43, p = 0.001). The proportion of participants in the overweight category (23.0-24.9 kg/m²) increased from 20.0% at baseline to 27.1% at 12 months. Mean serum creatinine increased from 0.88 ± 0.16 to 0.99 ± 0.21 mg/dL (mean increase = +0.11 mg/dL; t(69) = 4.86, p < 0.001); however, no participant developed renal impairment. Mean serum ALT rose from 30.3 ± 17.4 to 42.9 ± 34.9 IU/L (mean increase = +12.6 IU/L; Z = -5.97, p < 0.001); elevations were mild, with just one participant with hepatitis B co-infection developing a grade 3 ALT elevation attributed to immune reconstitution rather than drug-induced liver injury. Mean FBS increased from 95.0 ± 14.0 to 102.6 ± 32.7 mg/dL, which was statistically significant (t(69) = 2.00, p=0.049). No new diagnoses of diabetes mellitus occurred during follow-up. Metabolic, renal, and hepatic parameters are summarized in Table 2.

**Table 2:** Changes in metabolic, renal, and hepatic parameters in PLHIV initiated on TLD at 12 months (n = 70). ^#^: Wilcoxon signed rank test; *: Statistically significant at p < 0.05. PLHIV = people living with HIV; TLD = tenofovir disoproxil fumarate/lamivudine/dolutegravir; BMI = body mass index; ALT = alanine aminotransferase

Parameter	Baseline mean (SD)	12-month mean (SD)	Change from baseline (95% CI)	Test statistic	P-value
Weight (kg)	63.8 (12.5)	65.7 (13.1)	+1.89 (0.79–2.98)	t = 3.44	0.001*
BMI (kg/m²)	23.09 (3.84)	23.76 (3.94)	+0.67 (0.28–1.06)	t = 3.43	0.001*
Fasting blood sugar (mg/dL)	95.0 (14.0)	102.6 (32.7)	+7.6 (0.02–15.13)	t = 2.00	0.049*
ALT (IU/L)^#^	30.3 (17.4)	42.9 (34.9)	+12.6 (5.81–19.39)	Z = -5.97	<0.001*
Serum creatinine (mg/dL)	0.88 (0.16)	0.99 (0.21)	+0.11 (0.06–0.15)	t = 4.86	<0.001*

Correlation between changes in weight and body mass index with lipid levels

Spearman’s rank correlation analysis was performed to determine whether the lipid changes were associated with anthropometric changes. Change in body weight showed no significant correlation with change in any lipid parameter (Δ weight vs. ΔTC: ρ = 0.08, p = 0.491; vs. ΔLDL-c: ρ = 0.17, p = 0.162; vs. ΔHDL-c: ρ = 0.15, p = 0.221; vs. ΔTG: ρ = 0.11, p = 0.344). The mean change in BMI showed a significant though weak positive correlation with ΔHDL-c (ρ = 0.25, p = 0.040), and a borderline correlation between ΔBMI and ΔLDL-c (ρ = 0.23, p = 0.051), while ΔBMI was not significantly correlated with ΔTC (ρ = 0.21, p = 0.081) or ΔTG (ρ = 0.13, p = 0.267).

Association with demographic and anthropometric measures

At one year, incident dyslipidemia occurred in 31.0% (9/29) of participants aged 18-30 years, 29.4% (10/34) of those aged 31-50 years, and 71.4% (5/7) of those aged >50 years; the association with age category was not statistically significant (χ²(2) = 4.86; p = 0.088) but trended toward higher incidence in older participants. Dyslipidemia was observed in 34.6% of males (18/52) and 33.3% of females (6/18), without a statistically significant difference (Fisher’s exact test, p = 0.580); the limited female enrolment (n = 18) constrained statistical power for sex-stratified analysis. One of the six participants with a smoking history developed dyslipidemia. There was no significant association between smoking status and the development of dyslipidemia in this study population. (Fisher’s exact test, p = 0.415). Three of 12 participants with alcohol use had dyslipidemia. There was no significant association between alcohol intake and the development of dyslipidemia in this study population (Fisher’s exact test, p = 0.598).

## Discussion

In this prospective single-center cohort of 70 adult Indian PLHIV initiated on DTG-based cART, we observed a one-year incidence of dyslipidemia of 34.29%. The most frequent abnormalities were low HDL-c (24.29%) and hypertriglyceridemia (22.86%), followed by elevated LDL-c (12.86%) and elevated TC (7.14%). All lipid parameters except HDL-c showed statistically significant adverse mean changes; importantly, derived atherogenic indices, including non-HDL-c, TC/HDL-c ratio, and TG/HDL-c ratio, also worsened significantly, indicating that the lipid deterioration translated into a more atherogenic profile.

Our findings are consistent with those of the European RESPOND consortium, which reported a dyslipidemia incidence of 191.6 per 1,000 person-years among 4,577 PLHIV on contemporary cART, with DTG conferring an intermediate risk between PIs (lower) and rilpivirine (higher) [11]. Our 34.29% one-year incidence corresponds to approximately 343 per 1,000 person-years, almost double the RESPOND rate. The higher incidence in our study likely reflects differences in baseline cardiometabolic risk, the South Asian phenotype of central adiposity, and low HDL-c [21].

The dominance of low HDL-c and hypertriglyceridemia in our cohort closely parallels findings from sub-Saharan African DTG-based cohorts. Hamooya et al. reported low HDL-c in 75% of young Zambian adults on DTG-based ART [12], and a Ugandan cross-sectional study of 341 PLHIV on DTG-based ART found an overall prevalence of dyslipidemia of 78.0%, with low HDL-c in 72.1% and hypertriglyceridemia in 20.2% [22]. Collectively, these data identify low HDL-c as a characteristic feature of DTG-associated dyslipidemia, distinct from the LDL-c and TG-driven pattern historically associated with PIs.

Comparative lipid and metabolic effects of dolutegravir and other antiretroviral classes

The lipid signature of DTG in treatment-naïve initiation studies has generally been characterized as favorable in clinical trial settings. Saumoy et al., in a comprehensive review of randomized controlled trials of INSTIs, observed that DTG and raltegravir were consistently associated with smaller increases in TC, LDL-c, and TG than efavirenz- or boosted PI-based regimens, with modest, parallel increases in HDL-c and minimal change in the TC/HDL-c ratio. The choice of the nucleoside reverse transcriptase inhibitor (NRTI backbone, however, modifies this lipid signature: when DTG was combined with TAF or abacavir, slight increases in TC and LDL-c were observed, whereas when DTG was combined with TDF, lipid concentrations remained unchanged or showed a tendency to decline [10]. This pattern reflects the intrinsic lipid-lowering effect of TDF, originally demonstrated in the placebo-controlled TULIP trial [23], in which TDF-emtricitabine produced significant reductions in TC, LDL-c, and TG independent of any third-agent effect. The clinical implication is that the apparently favorable lipid profile of DTG in clinical trials is partly attributable to the TDF backbone in many of these studies, rather than to a uniformly lipid-neutral effect of DTG itself.

The integrase inhibitor class as a whole appears metabolically intermediate. A 2023 meta-analysis of six randomized controlled trials in ART-naïve PLHIV (n = 3,521) demonstrated that INSTI-based therapy was associated with a mean weight increase of 2.15 kg and small decreases in TC, LDL-c, HDL-c, and TG relative to PI- or NNRTI-based therapy [24]. The most clinically relevant moderator was the NRTI backbone: TAF accentuates weight gain and lipid increases, while TDF, the backbone in the Indian TLD regimen, exerts a counterbalancing lipid-lowering effect [23]. In a Swiss HIV Cohort Study, switching from TDF to TAF over 18 months produced significant increases in TC (+9.5 mg/dL), LDL-c (+4.7 mg/dL), and TG (+16.1 mg/dL) [25].

Against this backdrop, our real-world observation of significant increases in TC, LDL-c, and TG, alongside a decline in HDL-c and worsening atherogenic indices in a TDF-backbone cohort, is notable. Two factors may account for this divergence: first, baseline cardiometabolic risk in our predominantly overweight South Indian cohort (57.1% with BMI ≥23 kg/m² at baseline) likely differs from that of trial populations; second, the controlled dietary and lifestyle context of randomized trials may not reflect the real-world environment in which DTG-based ART is delivered through NACO programs. Therefore, the TDF lipid-lowering effect described by Saumoy et al. and Surial et al. may be insufficient to counterbalance the lipid perturbation associated with DTG initiation in populations with elevated baseline metabolic susceptibility [10,25].

Mechanisms of dolutegravir-associated dyslipidemia

In vitro and translational research has begun to characterize the mechanisms by which DTG modifies metabolism. Gorwood et al. demonstrated that DTG induces fibrosis, enhanced lipid accumulation, oxidative stress, mitochondrial dysfunction, and insulin resistance in human adipose stem cells and adipocytes [9]. These data collectively support a model in which DTG induces adipose tissue dysfunction that drives insulin resistance, weight gain, and secondary dyslipidemia, explaining why low HDL-c and elevated TG predominate over LDL-c elevation in DTG-treated cohorts.

We explored whether the observed lipid changes were associated with anthropometric changes over the 12-month follow-up period. While both BMI and lipid fractions worsened significantly over 12 months, change in body weight showed no meaningful correlation with change in any lipid parameter (all ρ < 0.17; all p > 0.16), and change in BMI was only weakly associated with change in HDL-c (ρ = 0.25, p = 0.040). Although this study was not designed to investigate causal mechanisms, these observations suggest that weight gain alone may not fully explain the observed lipid changes in this cohort and raise the possibility that additional mechanisms contribute.

Our findings differ from those of larger observational cohorts in which weight gain has been proposed as a potential mediator of antiretroviral-associated dyslipidemia. In the RESPOND consortium, adjustment for time-updated BMI attenuated the association between TAF exposure and dyslipidemia, suggesting that at least part of the excess lipid risk may be mediated through weight gain [11]. However, a random effect analysis comparing INSTI-based regimens with PI- or NNRTI-based regimens has reported small increases in weight gain but a decrease in lipids [24]. Several factors may explain the lack of a clear association in our study. First, our cohort was relatively small, limiting the power to detect modest correlations between anthropometric and metabolic changes. Second, participants were followed for only 12 months, and the metabolic consequences of weight gain may become more apparent with longer follow-up. Third, our study enrolled ART-naïve individuals initiating a TLD regimen, whereas much of the evidence linking weight gain and dyslipidemia derives from cohorts exposed to TAF-containing regimens, which appear to exert distinct effects on lipid metabolism.

Atherogenic indices and cardiovascular implications

A novel contribution of our analysis is the demonstration that derived atherogenic indices worsened significantly alongside individual lipid fractions. The TG/HDL-c ratio is a validated surrogate for insulin resistance, small, dense LDL particle predominance, and the atherogenic dyslipidemia of metabolic syndrome, and it predicts atherosclerotic cardiovascular disease independently of LDL-c. In a recent UK Biobank analysis of 342,979 participants, an elevated TG/HDL-c ratio was associated with a 60% increased risk of atherosclerotic coronary vascular disease at age 45 [26]. The TG/HDL-c ratio rose from 2.85 ± 1.06 at baseline to 4.04 ± 3.02 at 12 months (p = 0.002). Băneu et al. proposed sex-specific TG/HDL-c cut-offs of 2.53 for women and 2.80 for men as validated thresholds for insulin resistance and cardiometabolic dysfunction, derived from analyses spanning a range of body weights and age groups [27]. Notably, the mean baseline TG/HDL-c ratio in our cohort (2.85) already exceeded both sex-specific cut-offs even before DTG initiation, suggesting pre-existing insulin resistance in PLHIV that may reflect either the underlying chronic immune activation of HIV infection itself or the metabolic phenotype characteristic of the South Asian population. By 12 months, the ratio had risen to 4.04, placing the cohort well into a range associated with substantially elevated cardiovascular and metabolic disease risk. The fact that this ratio worsened significantly despite the cohort starting from an already-elevated baseline implies that DTG-based therapy compounded an underlying metabolic dysfunction already present in this population.

The TC/HDL-c ratio rose from 3.37 ± 0.82 at baseline to 4.03 ± 1.77 at 12 months (p = 0.029). The clinical significance of this increase is meaningful; in a CT coronary angiography study of 295 adults without coronary artery disease, Nair et al. demonstrated that individuals with an elevated TC/HDL-c ratio had a significantly higher prevalence of proximal coronary plaque (62% vs. 48%, p = 0.04) and a higher prevalence of significant coronary artery disease (19% vs. 9%, p = 0.009) compared with those lower ratios. In multivariate logistic regression, a TC/HDL-c ratio ≥4 emerged as an independent predictor of both significant coronary artery disease and proximal plaque burden [28]. The mean TC/HDL-c ratio in our cohort at 12 months crossed precisely the threshold (≥4) identified by Nair et al. as predictive of clinically significant coronary disease, despite the cohort being predominantly young (median age = 33 years). This shift from a mean baseline ratio of 3.37 to 4.03 within 12 months of treatment initiation suggests that DTG-based cART may rapidly transition PLHIV from a low-risk to a high-risk atherogenic phenotype, as defined by validated imaging-based cardiovascular endpoints.

Mean non-HDL-c rose significantly from 108.9 ± 32.3 to 119.8 ± 43.5 mg/dL (p = 0.014), approaching the ACC/AHA primary-prevention threshold of <130 mg/dL. Non-HDL-c has been shown in the ARIC study to capture residual atherogenic risk not reflected by LDL-c alone [29]. The simultaneous increases in the TC/HDL-c ratio (3.37 → 4.03), TG/HDL-c ratio (2.85 → 4.04), and non-HDL-c (+10.8 mg/dL) in our cohort suggest an overall worsening of cardiovascular risk that may not be fully captured by lipid thresholds alone.

Metabolic, renal, and hepatic findings

The mean weight gain (+1.89 kg) and BMI increase (+0.67 kg/m²) observed in our cohort are smaller than those reported in the ADVANCE trial (+4.3 kg with TLD over 96 weeks), likely reflecting differences in baseline BMI, ethnicity, and follow-up duration [7]. Although fasting blood sugar increased significantly over 12 months (mean increase = +7.6 mg/dL; t(69) = 2.00, p = 0.049), the magnitude of change was modest; no participant developed diabetes, and the clinical significance of this finding remains uncertain. The mild rise in creatinine (+0.11 mg/dL) is well characterized: DTG inhibits organic cation transporter 2 (OCT2)-mediated renal tubular secretion of creatinine without affecting true glomerular filtration, as confirmed by phase 1 studies using iohexol and para-aminohippurate clearances [30]. The increases in ALT we observed were predominantly mild; only one grade 3 elevation occurred, attributed to hepatitis B immune reconstitution rather than drug-induced liver injury, consistent with the favorable hepatic safety profile of DTG documented in clinical trials and real-world cohorts [5].

The Indian context and the evidence gap

This study builds on the limited published Indian literature. This study addresses three specific gaps in the existing Indian literature on DTG-associated dyslipidemia. First, all prior Indian studies have been limited to 24- to 26-week follow-up: Dravid et al.’s multi-center Phase IV trial of TLD in 250 treatment-naïve patients followed participants for 24 weeks and reported lipid changes as routine safety monitoring data without specific dyslipidemia incidence or stratification by lipid fraction [15]; Joshi et al. and Sekar et al. each followed predominantly ART-experienced switch populations for six months [16,17]. Our 12-month prospective follow-up captures lipid abnormalities that emerge beyond the early treatment period, a window not captured by prior Indian data. Second, our cohort comprised exclusively ART-naïve patients initiating the standard NACO TLD regimen, unlike Joshi et al.’s predominantly switch population (92.5% ART-experienced) or the heterogeneous regimens in Sekar et al.’s cohort, allowing isolation of the de novo lipid effects of TLD initiation without confounding from prior ART exposure. Third, we provide what is, to our knowledge, the first Indian analysis of atherogenic indices (TC/HDL-c ratio, TG/HDL-c ratio, non-HDL-c) in PLHIV on DTG, demonstrating that these composite markers, which are associated with imaging-confirmed coronary disease (TC/HDL-c ≥4), insulin resistance (TG/HDL-c >2.8), and atherosclerotic cardiovascular disease risk (non-HDL-c), worsened significantly within 12 months of TLD initiation. The convergence of our findings with the dyslipidemia signals documented by Joshi et al. and Sekar et al. strengthens the conclusion that DTG-associated lipid perturbation in Indian PLHIV is a real and reproducible phenomenon, not an artefact of any single cohort.

Implications for clinical practice

Our findings carry direct clinical implications in the post-REPRIEVE era. The REPRIEVE trial established that PLHIV derive cardiovascular benefit from statin therapy that is not predicted by conventional risk calculators, even at baseline LDL-c concentrations below the threshold for intervention in HIV-negative populations [13]. The 2026 ACC/AHA dyslipidemia guideline now recommends moderate-intensity statin therapy for PLHIV aged 40-75 years to mitigate cardiovascular risk [14]. Our data show that DTG-based cART produces clinically meaningful adverse changes in TC, LDL-c, TG, and atherogenic indices within 12 months of treatment initiation, accompanied by a significant increase in BMI. In aggregate, these findings argue for three clinical actions: first, frequent monitoring of fasting lipid profile, body weight, waist circumference, and atherogenic indices (TC/HDL-c, TG/HDL-c, non-HDL-c) for all PLHIV initiating DTG-based regimens; second, eligibility for primary prevention statin therapy should be assessed proactively per REPRIEVE-informed guidelines [14] with preference for pitavastatin, a non-CYP3A4-metabolized agent, as antiretroviral interactions are a concern; third, the operational implications for LMIC HIV programmes are substantial, given that scaling up primary prevention statin therapy to millions of PLHIV would require revisions to drug procurement, lipid-monitoring infrastructure, and counselling protocols. Our data quantify the magnitude of this need in the Indian context and provide the empirical foundation for such policy planning.

Strengths and limitations

Strengths of this study include its prospective design, 100% retention at 12 months, a formal a priori sample size calculation, use of pre-specified ATP III plus change-from-baseline criteria for incident dyslipidemia, comprehensive measurement of atherogenic indices and metabolic safety parameters, and focus on a treatment-naïve HIV population initiated on the NACO TLD regimen, most relevant to Indian clinical practice.

Limitations include the single-center design, which limits generalizability; the modest sample size, which was underpowered for secondary analyses and therefore these findings should be considered hypothesis-generating rather than confirmatory; 12-month follow-up duration, which precluded assessment of longer-term lipid trajectories and clinical cardiovascular events; absence of waist circumference, body composition, and visceral adipose tissue measurements; lack of routine viral load data for all participants; and absence of dietary and physical activity assessment, which may have influenced lipid outcomes. A multi-centrer, longer-term study incorporating these variables is warranted.

## Conclusions

In this prospective cohort of 70 adult Indian PLHIV initiated on the TDF-3TC-DTG regimen, the one-year incidence of dyslipidemia was 34.29%, characterized predominantly by low HDL-c (24.29%) and hypertriglyceridemia (22.86%). Mean TC, LDL-c, and TG all showed statistically significant adverse changes. Critically, derived atherogenic indices, TC/HDL-c ratio, TG/HDL-c ratio, and non-HDL-c, worsened significantly within 12 months of treatment initiation. The derived lipid indices crossed clinically validated cardiovascular risk thresholds in a predominantly young cohort without baseline dyslipidemia, indicating a meaningful transition toward an atherogenic phenotype despite short duration of ART exposure. Modest increases in BMI, FBS without incident diabetes, mild creatinine, and ALT elevations without kidney or drug-induced liver injury were also documented. These findings provide the first prospective Indian incidence estimate for dyslipidemia in PLHIV on the TDF-3TC-DTG regimen and underscore the need for structured lipid and metabolic surveillance to mitigate long-term cardiovascular risk in this expanding patient population.
